# Fatigue Induced by Physical and Mental Exertion Increases Perception of Effort and Impairs Subsequent Endurance Performance

**DOI:** 10.3389/fphys.2016.00587

**Published:** 2016-11-29

**Authors:** Benjamin Pageaux, Romuald Lepers

**Affiliations:** CAPS UMR1093, Institut National de la Santé et de la Recherche Médicale (INSERM), Université de Bourgogne-Franche ComtéDijon, France

**Keywords:** muscle fatigue, cognitive fatigue, cycling, running, time to exhaustion, time trial, aerobic exercise, perceived exertion

## Abstract

Endurance performance involves the prolonged maintenance of constant or self-regulated power/velocity or torque/force. While the impact of numerous determinants of endurance performance has been previously reviewed, the impact of fatigue on subsequent endurance performance still needs to be documented. This review aims to present the impact of fatigue induced by physical or mental exertion on subsequent endurance performance. For the purpose of this review, endurance performance refers to performance during whole-body or single-joint endurance exercise soliciting mainly the aerobic energy system. First, the impact of physical and mental exertion on force production capacity is presented, with specific emphasize on the fact that solely physical exertion and not mental exertion induces a decrease in force production capacity of the working muscles. Then, the negative impact of fatigue induced by physical exertion and mental exertion on subsequent endurance performance is highlighted based on experimental data. Perception of effort being identified as the variable altered by both prior physical exertion and mental exertion, future studies should investigate the underlying mechanisms increasing perception of effort overtime and in presence of fatigue during endurance exercise. Perception of effort should be considered not only as marker of exercise intensity, but also as a factor limiting endurance performance. Therefore, using a psychophysiological approach to explain the regulation of endurance performance would allow a better understanding of the interaction between physiological and psychological phenomena known to impact endurance performance.

## Introduction

Endurance performance involves the prolonged maintenance of constant or self-regulated power/velocity (e.g., Girard et al., 2012; Jones et al., 2016; Smits et al., 2016) or torque/force (e.g., Froyd et al., 2013; Pageaux et al., 2015a; Angius et al., 2016). Traditionally, endurance performance is measured by completion of time to exhaustion tests (i.e., open loop exercises) or time trials (i.e., closed loop exercises). While time to exhaustion tests consist in the maintenance of a fixed power/velocity or torque/force until exhaustion (i.e., disengagement from the exercise), time trials consist in the completion of a set amount of work as quickly as possible or as much work as possible in a set time. Both time to exhaustion tests and time trials have been shown to be reliable and valid measure of endurance performance (Laursen et al., 2007; Amann et al., 2008).

Endurance performance can be investigated via the use of whole-body exercises (e.g., cycling); or single-joint exercises (e.g., one leg dynamic exercise). While whole-body exercises present the advantage of replicating real sport events in laboratory conditions, single-joint exercises provide a unique exercise model to investigate underlying mechanisms thought to impact endurance performance. As an example, single-joint exercises present the advantage of reducing the time delay between the end of the endurance exercise and the start of neuromuscular testing (Pageaux et al., 2016). Therefore, researchers, coaches and athletes can benefit of considering both exercise models as a measure of endurance performance even if whole-body and single-joint exercises are known to induce different systemic responses to the exercise (Sidhu et al., 2013).

While the impact of numerous determinants (e.g., nutrition, oxygen uptake or sleep) of endurance performance has been previously reviewed (e.g., Bassett and Howley, 2000; Joyner and Coyle, 2008; McMahon et al., 2016; Simpson et al., 2016), the impact of fatigue on subsequent endurance performance still needs to be documented. The Oxford Dictionary defines fatigue as an “*extreme tiredness resulting from mental or physical exertion or illness*” and/or “*a reduction in the efficiency of a muscle or organ after prolonged activity.*” Therefore, the present review aims to present the impact of fatigue induced by physical or mental exertion on subsequent endurance performance. Firstly, the impact of physical and mental exertion on force production capacity will be presented, with specific emphasize on the fact that solely physical exertion and not mental exertion induces a decrease in force production capacity of the working muscles. Secondly, the negative impact of fatigue induced by physical exertion and mental exertion on subsequent endurance performance will be highlighted based on experimental data. Finally, as perception of effort during subsequent endurance exercise is the only variable altered by both prior physical and mental exertion, some insights on the impact of fatigue on perception of effort will be presented.

For the purpose of this review, endurance performance refers to performance during whole-body or single-joint endurance exercise soliciting mainly the aerobic energy system. Consequently, we considered only the studies that met the following criteria:

- The endurance exercise lasted at least 75 s (Gastin, 2001).
- Endurance performance was investigated as time to exhaustion tests, time trials or graded exercises.
- As this review focuses on the impact of fatigue on subsequent endurance performance, all studies included a physiological and/or psychological manipulation check attesting of the presence of fatigue prior to endurance performance measurement (see Table 1, “Markers of fatigue induced by the fatiguing protocol”).

**Table 1:** **Comprehensive list of studies investigating the impact of fatigue on subsequent endurance performance**.

**References**	**Subjects**	**Fatiguing protocol**	**Markers of fatigue induced by the fatiguing protocol**	**Endurance exercise**	**Impact of fatigue on endurance performance**	**RPE during the endurance performance test**
**FATIGUE OF A MUSCLE GROUP INVOLVED IN SUBSEQUENT WHOLE-BODY ENDURANCE EXERCISE**						
Amann and Dempsey, 2008	8 competitive ♂ cyclists, PL3	2 cycling conditions: (i) to exhaustion at 83% MAP, (ii) same duration at 67% MAP	↓ in KE MVC following 83% MAP, ↓ in KE twitch in both conditions, no change in VAL	5 km cycling time trial	↑ in time to complete the time trial, with a greater ↑ following 83% MAP	leg discomfort as a confounding factor
de Morree and Marcora, 2013	10 recreationally active ♂, PL2	100 drop-jumps (20 s rest between 2 jumps)	↓ in KE MVC no change in blood lactate	15 min cycling time trial	↓ in total work completed	↑
Deley et al., 2006	9 active ♂, PL2	2 conditions lasting 20 min: i) electromyostimulation, ii) voluntary contractions. KE isometric contractions 10 s ON—10 s OFF	↓ in KE MVC, twitch and VAL; greater ↓ in KE MVC and twitch post electromyostimulation	cycling time to exhaustion at 80% VO_2max_	↓ in time to exhaustion in both conditions, greater ↓ following electromyostimulation	not reported
Marcora et al., 2008	10 active ♂, PL2	100 drop-jumps (20 s rest between 2 jumps)	↓ in KE MVC, no change in KE muscle pain	cycling time to exhaustion at 80% MAP, ~90 ± 7% VO_2max_	↓ in time to exhaustion	↑
**FATIGUE OF A MUSCLE GROUP INVOLVED IN SUBSEQUENT SINGLE-JOINT ENDURANCE EXERCISE**						
Sherman et al., 1984	8 ♂runners, PL4	marathon	↓ in KE MVC	50 isokinetic KE concentric contractions	↓ in total work completed	not reported
Neyroud et al., 2012	14 physically active ♂, no information for PL determination	20% KE MVC time to exhaustion	↓ in KE MVC, twitch and VAL	20% KE MVC time to exhaustion	↓ in time to exhaustion	↑
**FATIGUE OF A MUSCLE GROUP NON-INVOLVED IN SUBSEQUENT WHOLE-BODY ENDURANCE EXERCISE**						
Johnson et al., 2014	7 moderately trained ♂, PL2	8 × 1 min interspaced by 30 s rest at 1.5–2.0 W/kg	↑ in blood lactate and ion hydrogen, no measurement of force production capacity	Incremental cycling test, time to exhaustion at 85% MAP CP and W' estimation	↓ MAP and VO_2max_ achieved during the incremental cycling test, ↓ in time to exhaustion and W', no change in CP	not reported
Johnson et al., 2015	8 moderately trained ♂, PL2	8 × 1 min interspaced by 30 s rest at 1.0–1.5 W/kg	No measurement of force production capacity of the upper limbs, ↑ in blood lactate	time to exhaustion at 85% MAP	↓ in time to exhaustion, greater ↓ in KE MVC following the control time to exhaustion test	leg discomfort as a confounding factor
**FATIGUE OF A MUSCLE GROUP NON-INVOLVED IN SUBSEQUENT SINGLE-JOINT ENDURANCE EXERCISE**						
Amann et al., 2013	8 recreationally active ♂, PL2	unilateral KE isotonic contractions at 85% MAP to exhaustion	↓ in KE MVC of the pre fatigued leg, ↓ in KE twitch of the pre fatigued leg, no change in VAL	controlateral KE isotonic contractions at 85% MAP to exhaustion	↓ in time to exhaustion	↑
Bangsbo et al., 1996	7 active ♂, PL2	4 × 1 min arm cranking at 137 ± 3 W	↑ in muscle lactate, no measurement of force production capacity	KE isotonic contractions at 61.4 ± 3.7 W to exhaustion	↓ in time to exhaustion	not reported
Nordsborg et al., 2003	6 active ♂, PL2	4 × 1 min arm cranking at ~ 140 W	↑ in interstitial potassium, no measurement of force production capacity	KE isotonic contractions at 62.8 ± 3.0 W to exhaustion	↓ in time to exhaustion	not reported
Triscott et al., 2008	3 groups of 8 subjects: sedentary (PL1), resistance (PL2–3), endurance (PL2–3)	unilateral bicep curls to exhaustion (weight 5.5 kg)	↓ in EF MVC of the pre fatigued arm	controlateral bicep curls to exhaustion (weight 4.5 kg)	↓ in time to exhaustion	not reported
**FATIGUE INDUCED BY MENTAL EXERTION AND SUBSEQUENT WHOLE-BODY ENDURANCE EXERCISE**						
MacMahon et al., 2014	18 trained ♂ and 2 trained ♀, PL2	90 min of AX-continuous performance test	↑ in heart rate during the cognitive task, ↑ in self-reported fatigue, no measurement of force production capacity	3 km running time trial	↑ in time to complete the time trial	↑ (same RPE for a lower running velocity)
Marcora et al., 2009	10 active ♂ and 6 active ♀, PL2	90 min of AX-continuous performance test	↑ in heart rate during the cognitive task, ↑ in self-reported fatigue, ↓ in cognitive performance, no measurement of force production capacity	time to exhaustion at 80% MAP	↓ in time to exhaustion	↑
Martin et al., 2016	11 professional ♂ road cyclists (PL5) and 9 recreational ♂ cyclists (PL1–2)	30 min of incongruent Stroop task	↑ in self-reported fatigue for both groups, no measurement of force production capacity	20 min cycling time trial	↓ in power output in the PL 1–2 group only no change in performance in the PL 5 group	↑ in the PL1–2 group (same RPE for lower power output) no change in the PL 5 group
Pageaux et al., 2014	10 recreationally active ♂, PL2	30 min of incongruent Stroop task	↑ in heart rate during the cognitive task, ↑ in mental demand and effort, no measurement of force production capacity	5 km running time trial	↑ in time to complete the time trial	↑
Smith et al., 2015	10 recreationally active ♂, PL2	90 min of AX-continuous performance test	↑ in heart rate during the cognitive task, ↑ in self-reported fatigue, no measurement of force production capacity	45 min self-paced intermittent running protocol replicating team sports physical demand	↓ in running velocity	↑ (same RPE for a lower running velocity)
Smith et al., 2016	12 moderately trained soccer ♂, PL2	30 min of incongruent Stroop task	↑ in self-reported fatigue, no measurement of force production capacity	Yo-Yo Intermittent Recovery Test, Level 1	↓ in running distance	↑
**FATIGUE INDUCED BY MENTAL EXERTION AND SUBSEQUENT SINGLE-JOINT ENDURANCE EXERCISE**						
Pageaux et al., 2013	10 active ♂, PL2	90 min of AX-continuous performance test	↑ in heart rate during the cognitive task, ↑ in self-reported fatigue, no change in KE MVC	20% KE MVC time to exhaustion	↓ in time to exhaustion	↑

All studies presented in this table are discussed within the manuscript. EF, elbow flexors; KE, knee extensors; MVC, maximal voluntary contraction; PL, performance level (De Pauw et al., 2013); RPE, ratings of perceived exertion; VAL, voluntary activation level. ↑, increase; ↓, decrease; MAP, maximal aerobic power; CP, critical power.

A comprehensive list of studies included in this review is presented in Table 1.

## Fatigue induced by physical exertion and mental exertion: impact on force production capacity

When completion of physical exertion induces a reduction in force production capacity of a muscle group, fatigue is traditionally defined as muscle fatigue (Gandevia, 2001). When completion of mental exertion induces a reduction in cognitive performance and/or an increase in subjective feelings of tiredness and lack of energy, fatigue is traditionally defined as mental fatigue (Boksem and Tops, 2008).

Fatigue induced by physical exertion has been extensively studied in the literature (for review please see Enoka and Stuart, 1992; Gandevia, 2001; Enoka and Duchateau, 2008) and could be investigated by measuring the maximal force, torque or power that can be produced by a muscle or a muscle group. A reduction in maximal force, torque or power obtained during maximal voluntary contractions (MVC) is the gold standard to identify the presence of reduced force production capacity (Gandevia, 2001). This reduction in force production capacity has been shown to be caused by an inability of the central nervous system to maximally recruit the working muscles (i.e., traditionally defined as central fatigue; Gandevia, 2001) and also by changes at or distal to the neuromuscular junction, impairing contractile properties of the working muscles (i.e., traditionally defined as peripheral fatigue; Allen et al., 2008).

Fatigue induced by mental exertion is a psychobiological state caused by prolonged engagement in mentally demanding cognitive activities (Boksem and Tops, 2008). Its presence is traditionally identified by completion of questionnaires allowing the athlete/subject to report his/her feelings of fatigue, defined as tiredness and lack of energy (Boksem and Tops, 2008). This methodology has been shown to be successful in identifying presence of fatigue induced by mental exertion lasting at least 30 min (e.g., Marcora et al., 2009; Pageaux et al., 2013; Smith et al., 2016). Interestingly, as fatigue induced by mental exertion (Wang et al., 2016) and central fatigue (Taylor et al., 2000) are both phenomena occurring in brain areas upstream of the primary motor cortex, several authors have proposed an association between these two phenomena (e.g., Newsholme et al., 1992; Di Giulio et al., 2006). However, recent studies focusing on the impact of mental exertion on force production capacity demonstrated that mental exertion does not impair the ability of an athlete/subject to maximally recruit his/her working muscles (Pageaux et al., 2013, 2015b; Martin et al., 2014; Rozand et al., 2014; Duncan et al., 2015). Therefore, as only fatigue induced by physical exertion and not by mental exertion impairs force production capacity (Pageaux et al., 2015b), it seems crucial to differentiate the kind of exercise inducing fatigue.

## Fatigue of a muscle group involved in subsequent endurance exercise impairs endurance performance

### Whole-body exercise

In 2006, Deley and colleagues tested the impact of a decrease in knee extensors (KE) force production capacity, using either electromyostimulation or voluntary contractions, on the VO_2_ kinetics during a cycling time to exhaustion test performed at 80% VO_2max_. These authors demonstrated not only that the appearance of the VO_2_ slow component was delayed in the electromyostimulation condition and its amplitude was lower than that obtained in the voluntary contractions condition, but also that endurance performance was reduced in both fatiguing conditions compared to the control condition. In addition, the decrease in endurance performance was greater following electromyostimulation (−25.9%) compared to voluntary contractions (−6.4%), thus in relation to the extent of KE MVC reduction (electromyostimulation: −19.9%, voluntary contractions: −11.8%). To the best of our knowledge, this study is the first to report a decrease in endurance performance induced by fatigue of a muscle group involved in subsequent endurance exercise. Similar results were observed by Amann and Dempsey (2008) with a different fatiguing protocol. In this study, a reduction in KE MVC was induced by either a cycling time to exhaustion test at 83% of maximal aerobic power (MAP) or a cycling exercise of the same duration at 67% MAP. Both fatiguing conditions induced an increase in time to complete the subsequent 5 km cycling time trial, with a greater increase following the 83% MAP condition (+6%) compared to the 67% MAP condition (+2%). This greater increase in time to complete the 5 km cycling time trial in the 83% MAP condition was associated with a greater extent of KE MVC reduction post time to exhaustion test at 83% MAP compared to cycling for the same duration at 67% MAP. However, it has to be noticed that none of the two studies aforementioned controlled for the confounding factor of prior exercise induced accumulation of metabolites, thought to influence endurance performance (Amann, 2011). For this reason, Marcora and colleagues (Marcora et al., 2008; de Morree and Marcora, 2013) performed similar experiments by looking at the effects of a fatigue protocol known to induce a significant reduction in KE force production capacity in absence of significant accumulation of muscle metabolites (Skurvydas et al., 2000, 2002). Marcora and colleagues confirmed the results of previous studies by demonstrating that even without accumulation of muscle metabolites; a reduction in KE force production capacity induces a decrease in subsequent cycling endurance performance. Therefore, when integrating the results of the studies aforementioned, it is clear that fatigue of a muscle group involved in subsequent whole-body endurance exercise decreases endurance performance.

### Single-joint exercise

In 1984, Sherman and colleagues demonstrated that completion of a marathon decreases the amount of work performed during a work capacity test consisting in the repetition of 50 maximal leg extensions (duration of the endurance exercise ~2 min). This impairment in endurance performance was confirmed by Neyroud et al. (2012). In this study, the duration of a continuous KE isometric contraction at 20% MVC to exhaustion was reduced by 70% when performed subsequently to an initial KE isometric contraction at 20% MVC to exhaustion. Therefore, as previously discussed for whole-body endurance exercise, fatigue of a muscle group involved in subsequent single-joint endurance exercise also decreases endurance performance.

## Fatigue of a muscle group non-involved in subsequent endurance exercise impairs endurance performance

### Whole-body exercise

To the best of our knowledge, only Johnson and colleagues investigated the impact of fatigue of a muscle group non-involved in subsequent whole-body endurance exercise on endurance performance (Johnson et al., 2014, 2015). To do so, the authors performed intermittent arm cranking to fatigue the upper limbs, and then investigated the impact of this previous exercise on subsequent cycling endurance performance. The authors demonstrated that even if the arm cranking exercise does not alter subsequent critical power estimation (Johnson et al., 2014), arm cranking impairs subsequent cycling endurance performance. This decrease in cycling endurance performance was evidenced by a decrease in MAP (−7%) and VO_2max_ (−14%) achieved during a graded exercise (Johnson et al., 2014), and a decrease in time to exhaustion at 85% MAP (~35%; Johnson et al., 2014, 2015).

### Single-joint exercise

When endurance performance is investigated with single-joint exercise, the impact of fatigue of a muscle group non-involved in subsequent endurance exercise on endurance performance could be investigated by either fatiguing one limb and testing the controlateral limb endurance performance, or by fatiguing the upper body (or lower body) and testing a lower body (or upper body) muscle group endurance performance. With regard to endurance performance of the controlateral limb, Amann et al. (2013) demonstrated a decrease in controlateral KE isotonic contractions time to exhaustion performed at 85% MAP (−49%) following a previous unilateral KE isotonic contractions time to exhaustion performed at same intensity. In 2008, Triscott and colleagues demonstrated a ~20% decrease in controlateral biceps curls time to exhaustion following unilateral biceps curls to exhaustion. In both studies, neither the unilateral biceps curls time to exhaustion nor the unilateral KE isotonic contractions time to exhaustion induced a reduction in force production capacity of the controlateral limb involved in the subsequent endurance exercise. With regard to subsequent endurance performance of a lower body muscle group following prior fatiguing exercise involving the upper body, Bangsbo et al. (1996) and Nordsborg et al. (2003) demonstrated a negative impact of prior arm cranking on KE isotonic contractions time to exhaustion. By using identical fatiguing protocol (4 × 1 arm cranking at ~140 W), the authors observed a 26% (Bangsbo et al., 1996) and 32% (Nordsborg et al., 2003) decrease in KE isotonic contractions time to exhaustion at ~60 W.

## Fatigue induced by mental exertion impairs endurance performance

### Whole-body exercise

Since the first study of Marcora et al. (2009) demonstrating an impairment of 15% in cycling endurance performance caused by prior mental exertion, numerous studies investigating the impact of fatigue induced by mental exertion on whole-body endurance performance have been published (MacMahon et al., 2014; Pageaux et al., 2014; Smith et al., 2015, 2016). All these studies reached a consensus on the negative impact of prior mental exertion on endurance performance; even so elite athletes present a greater resistance to fatigue induced by prior mental exertion (Martin et al., 2016). This impairment was observed during cycling (Marcora et al., 2009; Martin et al., 2016) and running exercises (MacMahon et al., 2014; Pageaux et al., 2014). Interestingly, Smith and colleagues demonstrated that fatigue induced by mental exertion also impairs prolonged intermittent (Smith et al., 2015) and graded (Smith et al., 2016) running exercises. Consequently, it seems clear that fatigue induced by mental exertion decreases endurance performance, even if prior mental exertion does not alter physiological responses to endurance exercise (Marcora et al., 2009; Pageaux et al., 2013).

### Single-joint exercises

To the best of our knowledge, only one study investigated the impact of prior mental exertion on endurance performance. In this study, Pageaux et al. (2013) demonstrated that even if prior mental exertion does not reduce KE force production capacity, fatigue induced by mental exertion still causes a decrease in endurance performance during a continuous KE isometric contraction at 20% MVC to exhaustion. However, no study investigated the impact of fatigue induced by mental exertion on single-joint time trial.

## Prior physical exertion and prior mental exertion increase perception of effort during subsequent endurance exercise

**Figure 1:**
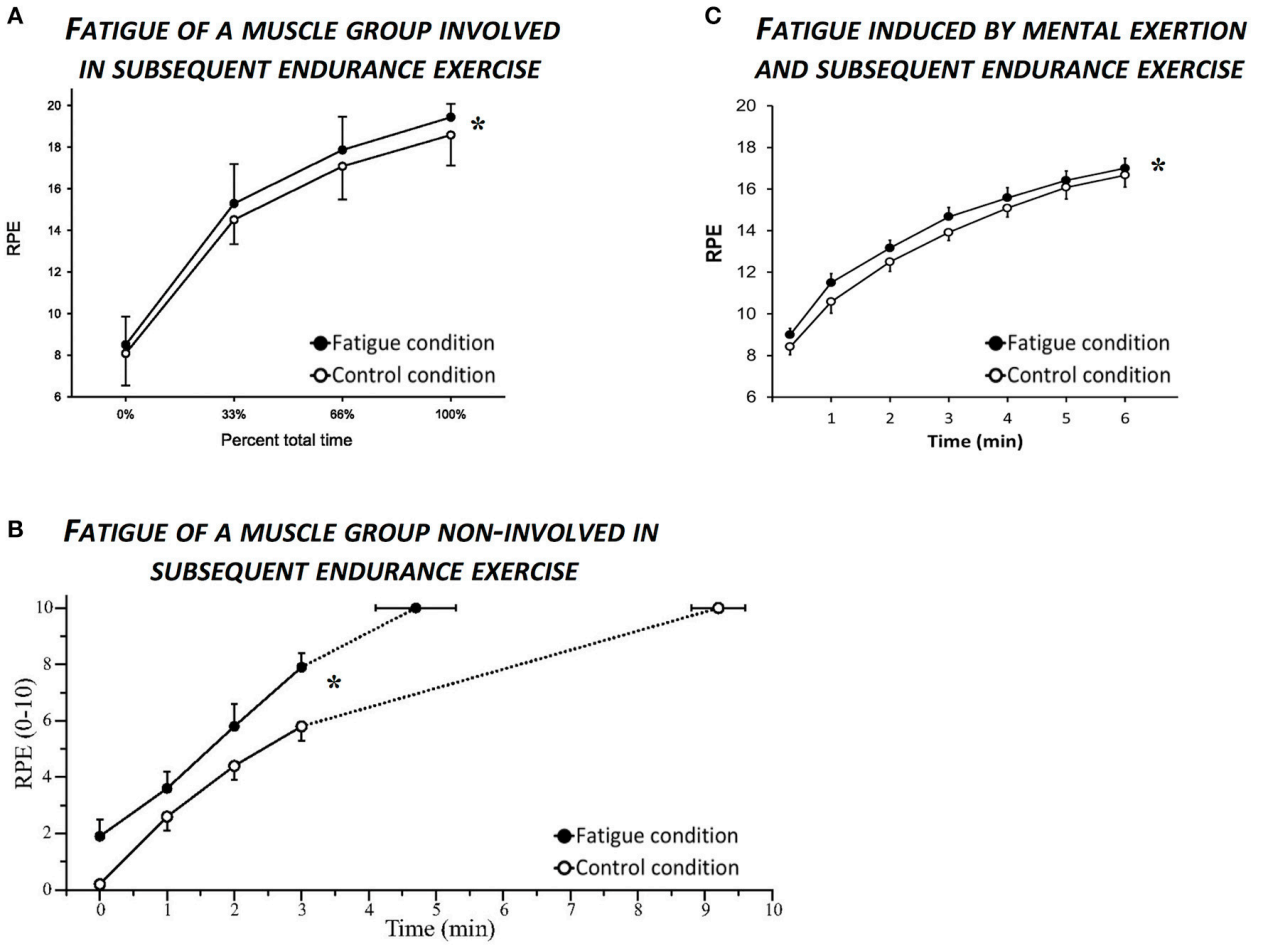
**Effects of fatigue induced by physical and mental exertion on ratings of perceived exertion (RPE) during subsequent endurance exercise. (A)** Illustrates the increase in RPE induced by fatigue of a muscle group (knee extensors) involved in subsequent endurance exercise (constant load cycling exercise at 80% maximal aerobic power). Reprinted with permission and adapted from Marcora et al. (2008), p. R880, Figure 6A. **(B)** Illustrates the increase in RPE induced by fatigue of a muscle group (knee extensors) non-involved in subsequent endurance exercise (controlateral isotonic knee extension at 85% maximal aerobic power). Reprinted with permission and adapted from Amann et al. (2013), p. 361, Figure 4. **(C)** Illustrates the increase in RPE caused by Stroop task-induced fatigue during subsequent endurance exercise (constant load cycling exercise at 80% maximal aerobic power). Adapted from Pageaux et al. (2015b), p. 8, Figure 4A. In the three studies, RPE was higher during the fatigue condition compared to the control condition, as illustrated with the ^*^ representing a significant effect of condition (*p* < 0.05).

As demonstrated by the studies included in this review (see Table 1), endurance performance could be altered in absence (e.g., Pageaux et al., 2013) or presence (e.g., Marcora et al., 2008) of a decrease in force production capacity of the working muscles involved in the subsequent exercise. Endurance performance could also be altered with (e.g., Amann et al., 2013) or without (e.g., Marcora et al., 2009) altered physiological responses to the exercise. Therefore, these results raise a simple question: do physical exertion and mental exertion alter a common variable during subsequent endurance exercise? As shown in Figure 1, the answer is yes. This variable altered by both physical exertion and mental exertion is the perception of effort.

Perception of effort (also referred as perceived exertion or sense of effort), defined as “the feeling of how hard, heavy and strenuous a physical task is” (Marcora, 2010; Pageaux, 2016), is a cognitive feeling of work associated with voluntary actions (Preston and Wegner, 2009; Pageaux, 2016). This perception differs from other exercise-related sensations such as pain or discomfort (Pageaux, 2016), and can be rated via the use of psychophysiological scales such as the Borg ratings of perceived exertion scale or the category ratio (CR)10 scale (Borg, 1998). While a persistent debate exists in the literature on the neurophysiology of perceived exertion (Marcora, 2009; Pageaux, 2016), it exists strong experimental data providing evidence that perception of effort results from the neuronal process of the corollary discharge associated with the central motor command (Marcora, 2009; Pageaux, 2016; Pageaux and Gaveau, 2016). Indeed, studies using pharmacological blockade of muscle afferents demonstrated that in absence of muscle afferent feedback, perception of effort is not reduced during endurance exercise (Pageaux and Gaveau, 2016). While the increased perceived exertion in presence of fatigue of a muscle group involved in subsequent endurance exercise is associated with an increase in activity of cortical premotor and motor areas (i.e., index of central motor command) to compensate for alteration of neuromuscular properties of the working muscles (de Morree et al., 2012); the underlying mechanisms behind the increased perceived exertion induced by fatigue of a muscle group non-involved in subsequent endurance exercise and fatigue induced by mental exertion remain unclear. Although some authors proposed an increase in afferent feedback caused by fatigue of a muscle group non-involved in subsequent endurance exercise to be responsible of the increased perceived exertion (Amann et al., 2013), this hypothesis is unlikely because spinal blockade of muscle afferents does not reduce perceived exertion (Pageaux and Gaveau, 2016). An alternative hypothesis could be that prior prolonged activation of premotor and motor areas associated with the completion of the fatiguing exercise would induce intrinsic changes in the brain, inducing an alteration of the activation of premotor and motor areas in the subsequent exercise. As prolonged neural activity has been shown in animal studies to increase extracellular concentrations of adenosine (Lovatt et al., 2012), an increase in extracellular concentrations of adenosine caused by prior physical exertion could be a good candidate to explain the increased perceived exertion caused by fatigue of a muscle group non-involved in subsequent endurance exercise. This hypothesis has also been proposed to explain the increased perceived exertion caused by prior mental exertion (Pageaux et al., 2014, 2015b), and find additional support with (i) studies demonstrating a positive impact of caffeine (i.e., an antagonist of adenosine) ingestion on physical and cognitive performances (McLellan et al., 2016); and (ii) recent experimental evidence demonstrating the involvement of premotor and motor areas in cognition and decision-making process (Morsella et al., 2015; Ramkumar et al., 2016; Tomasino and Gremese, 2016). Therefore, future studies should investigate the underlying mechanisms responsible of the increased perception of effort during exercise and caused by prior physical and mental exertion.

By integrating experimental results from different exercise modes and published by different research groups, this review provides evidence that fatigue induced by prior physical or mental exertion impairs subsequent endurance performance. While impairments in endurance performance are not associated with a common physiological alteration, perceived exertion seems to be the common variable altered by fatigue. Consequently, future studies should investigate the cause of the progressive increase in perceived exertion overtime during endurance exercise and consider perceived exertion not only as marker of exercise intensity, but also as a factor limiting endurance performance. Furthermore, as psychological interventions such as self-talk (Blanchfield et al., 2013) or subliminal images (Blanchfield et al., 2014) could be used to manipulate endurance performance; special attention should be given to models aiming to explain regulation of endurance performance with a psychophysiological approach (Marcora et al., 2008; Millet, 2011; Pageaux, 2014). Using a psychophysiological approach would allow a better understanding of the interaction between physiological and psychological phenomena known to impact endurance performance.

## Author contributions

Both authors have approved the final version of the manuscript and agree to be accountable for all aspects of the work.

## Funding

The manuscript has been written during the postdoctoral position of BP funded by the Région de Bourgogne (contract 9201AAO050S02953) and the Fonds Européen de Développement Régional (FEDER). The funders of the postdoctoral position had no role in decision to publish, or preparation of the manuscript.

### Conflict of interest statement

The authors declare that the research was conducted in the absence of any commercial or financial relationships that could be construed as a potential conflict of interest.
